# LDPC-Coded CAP with Spatial Diversity for UVLC Systems over Generalized-Gamma Fading Channel

**DOI:** 10.3390/s20123378

**Published:** 2020-06-15

**Authors:** Hongyan Jiang, Hongbing Qiu, Ning He, Zhonghua Zhao, Wasiu Popoola, Zahir Ahmad, Sujan Rajbhandari

**Affiliations:** 1 School of Information and Communication, Guilin University of Electronic Technology, Guilin 541004, China; jianghy@guet.edu.cn (H.J.); qiuhb@guet.edu.cn (H.Q.); eicnhe@guet.edu.cn (N.H.); gietzzh@guet.edu.cn (Z.Z.); 2 Guangxi Key Laboratory of Wireless Wideband communication and Signal Processing, Guilin 541004, China; 3 School of Engineering, Institute for Digital Communications, University of Edinburgh, Edinburgh EH8 9YL, UK; w.popoola@ed.ac.uk; 4 School of Computing, Electronics and Mathematics, Coventry University, Coventry CV1 5FB, UK; ab7175@coventry.ac.uk; 5 Huawei Technologies Sweden AB, Gothenburg 41250, Sweden

**Keywords:** UVLC, CAP, LDPC, turbulence-induced fading, GG distribution

## Abstract

In this paper, low-density parity-check (LDPC)-coded carrierless amplitude and phase (CAP) modulation with spatial diversity is proposed to mitigate turbulence-induced fading in an underwater visible-light communication (UVLC) channel. Generalized-gamma (GG) distribution was used to model the fading, as this model is valid for weak- and strong-turbulence regimes. On the basis of the characteristic function (CHF) of GG random variables, we derived an approximated bit-error rate (BER) for the CAP modulation scheme with spatial diversity and equal-gain combining (EGC). Furthermore, we simulated the performance of the CAP system with diversity and LDPC for various turbulence conditions and validated the analysis. Obtained results showed that the combination of LDPC and spatial diversity is effective in mitigating turbulence-induced fading, especially when turbulence strength is strong.

## 1. Introduction

With increasing oceanic activities, such as environmental monitoring, resource exploration, marine archaeology, and oceanographic-data collection, there has been huge demand for high-speed reliable short-to-medium-range underwater communication [1]. Recently, underwater visible-light communication (UVLC) has drawn significant attention due to the availability of high-bandwidth, high-speed data communication, and low cost [2]. However, the performance of a UVLC system is adversely affected by absorption, scattering, and underwater optical turbulence (UOT). Hence, to realise the full potential of UVLC, appropriate technique(s) should be adopted to alleviate these impairments [3]. Among these impairments, absorption and scattering were extensively studied [4,5,6]. UOT results from changes in the refractive index of water caused by random variations of water temperature and salinity, and the presence of air bubbles that result in random fluctuations on the received signal known as turbulence-induced fading. The performance of underwater optical wireless communication systems in the presence of underwater turbulence is often modelled with the log-normal distribution [2,3,7,8]. This is similar to the model used for weak atmospheric turbulence [9]. Recently, there have been various efforts to accurately model oceanic-turbulence-induced fading [1,10,11]. It was shown that generalized-gamma (GG) and exponential Weibull distributions can excellently model fading induced by weak-to-strong UOT [11]. In fact, it was shown that many important statistical distributions to model optical turbulence, such as Weibull, gamma, Nakagami-*m*, exponential, and Rayleigh, are special cases of GG distribution [12].

To mitigate the effects of turbulence-induced fading, several techniques, such as forward-error correction (FEC) [13], diversity [2,3,8], and multihop relaying [7], were suggested. For short-range communication, coding and diversity are preferred due to low complexity and ease of implementation. Low-density parity-check (LDPC) code is a highly efficient linear block code characterised by a sparse parity-check matrix that results in low hardware complexity. The error-correction performance of LDPC can approach the Shannon limit in an additive white Gaussian noise (AWGN) channel [14]. LDPC codes provide higher code gain, and perform significantly better than turbo-products and Reed–Solomon codes [13,15]. Furthermore, the decoding complexity of LDPC codes increases linearly in comparison to the exponential increment in turbo code. Thus, LDPC has been widely employed in optical communication [13,16]. Meanwhile, spatial diversity is considered as one of the most effective solutions to mitigate turbulence-induced fading. Spatial diversity also decreases the possibility of temporary blockage due to an obstruction (e.g., fish) [8]. Additionally, the selection of the modulation scheme is equally vital for the optimal performance of UVLC systems. Carrierless amplitude and phase (CAP) modulation is an attractive scheme in UVLC due to simple implementation, low energy consumption, and high spectral efficiency [17]. CAP modulation is more suitable to intensity-modulation and direct-detection (IM/DD)-based optical communication than its counterpart, quadrature-amplitude modulation (QAM), as the CAP is realised using a pair of orthogonal filters to transmit two parallel streams of data without a carrier [18]. In addition, CAP has similar spectral efficiency as that of orthogonal frequency division multiplexing (OFDM) with a lower peak-to-average-power-ratio (PAPR) than that of OFDM [19].

This analysis suggests that combining efficient modulation with effective FEC and diversity offers optimal performance for UVLC systems. Hence, in this study, we propose an LDPC-coded CAP modulation scheme with spatial diversity to improve the performance of UVLC systems over a UOT-induced fading channel. In this study, the LDPC outlined in IEEE.802.16 standard, whose fundamental codes can accommodate various code rates and packet sizes [20], was adopted. The UOT fading channel was modelled by GG distribution covering from weak to strong turbulence. We derived closed-form expressions of the approximated bit-error rate (BER) for the UVLC spatial-diversity scheme with the equal-gain combining (EGC) at the receiver on the basis of the characteristic function (CHF) of GG random variables. Then, we used Monte Carlo (MC) simulations to investigate the various spatial-diversity orders in combination with LDPC, validating theoretical analysis and the effect to combat UOT. To the best of the authors’ knowledge, this is the first study providing comprehensive mathematical derivation validated by MC simulations to evaluate the performance of the LDPC-coded spatial diversity CAP scheme over GG-distributed UVLC fading channels, which is helpful to design a reliable UVLC system in turbulent channels.

The remainder of the paper is organised as follows. In Section 2, the UVLC system, fading channel model, and LDPC selection are described. In Section 3, we derived the approximated BER on the basis of the CHF of GG random variables. Then, the Monte Carlo simulation is carried out in various channel scenarios, and the results are presented in Section 4. Lastly, Section 5 concludes the paper.

## 2. CAP-Based UVLC System

### 2.1. System Model

**Figure 1:**
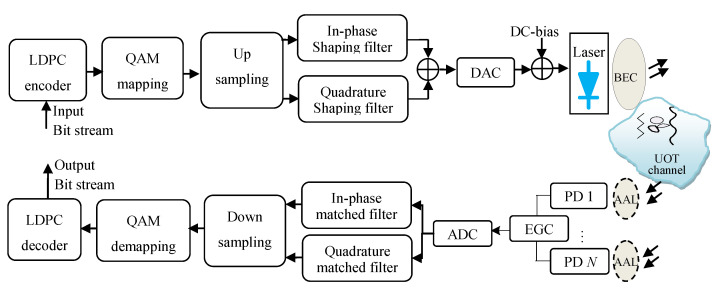
Block diagram of spatial-diversity underwater visible-light communication (UVLC) system employing carrierless amplitude and phase (CAP) modulation scheme and low-density parity-check (LDPC) codes.

Figure 1 shows a simplified block diagram of the LDPC-coded CAP modulation scheme with spatial diversity at the receiver. The system consisted of one transmitter and *N* receivers with the same aperture size. The pseudorandom binary streams were parsed into groups of *k*-bits and encoded using LDPC code, which resulted in corresponding groups of codewords with *n*-bits, where *k* and *n* were the information length and code length of LDPC, respectively. The *n*-bits in each codeword were then mapped into an *M*-QAM signal using a Gray mapping rule. The QAM signal was upsampled by a factor of *N_s_* followed by separation of inphase (I) and quadrature-phase (Q) sequences, and application of two orthogonal pulse-shaping filters. The impulse responses of the orthogonal filters were given by the multiplication of cosine and sine with a root-raised cosine filter (RRCF) for I and Q signals, respectively, i.e., the impulse responses of pulse shaping filters were given by [21,22].

(1) $$f_{I}\left(t\right)=g\left(t\right)\cos\left(2\pi f_{c}t\right)$$

(2) $$f_{Q}\left(t\right)=g\left(t\right)\sin\left(2\pi f_{c}t\right),$$

where *f_c_* is the carrier frequency, and g(t) is the RRCF, given by

(3) $$g\left(t\right)=\frac{\sin\left[\frac{\pi t}{T}\left(1-\beta \right)\right]+\frac{4\beta t}{T}\cos\left[\frac{\pi \left(1+\beta \right)t}{T}\right]}{\frac{\pi t}{T}\left[1-{\left(\frac{4\beta t}{T}\right)}^{2}\right]}$$

where *T* is the symbol duration, and *β* is the roll-off factor of RRCF.

The filter outputs were then summed, i.e.,

(4) $$S\left(t\right)=A_{I}\left(t\right)⊗f_{I}\left(t\right)-A_{Q}\left(t\right)⊗f_{Q}\left(t\right)$$

where ⊗ represents time-domain convolution operation, and $\;A_{I}\left(t\right)$ and $A_{Q}\left(t\right)$ denote in-phase and quadrature *M*-QAM symbols.

The signal was then converted to an analogue signal by a digital-to-analogue converter (DAC). Since the IM/DD requires a positive signal, DC bias $I_{\mathrm{DC}}$ was added to bipolar CAP signals. The DC bias was given by [23]

(5) $$I_{\mathrm{DC}}=g\sqrt{E_{s}}$$

Where g is the normalised bias, and $E_{s}$ is the energy per symbol.

The modulated beam was expanded and collimated to ease alignment and sufficiently illuminate all receiver apertures. After going through the turbulence channel (details of turbulence channel are given in the following section), the beam was received by spatial-diversity receivers. We assumed that the spatial-correlation width was larger than the aperture diameter of each receiver, and the receiver separations were sufficiently larger than coherence length, whose typical value is on the order of millimeters [24,25]. Thus, the signal at each receiver branch was independent, and the received instantaneous electrical signal with EGC was given by

(6) $$y\left(t\right)=\eta x\left(t\right)Ps\overset{N}{\underset{i=1}{\sum }}\alpha_{i}h_{i}+\overset{N}{\underset{i=1}{\sum }}n_{i}\left(t\right)$$

where *η* denotes photodetector responsivity, x(t) represents the modulated symbol, Ps is the irradiance at the receiver, and *α_i_*, $h_{i}$, and $n_{i}\left(t\right)$ represent the fading coefficient, path loss, and additive white Gaussian noise (AWGN) of the *i*^th^ branch, respectively. We assumed that path loss $h_{i}$ was independent of the fading process and normalised to unity. Meanwhile, all receivers had the same noise variance of *σ*^2^. Thus, the electrical signal-to-noise ratio (SNR) of EGC was given by

(7) $$\gamma_{\mathrm{EGC}}=\frac{{\left(\eta Ps\right)}^{2}E\left[x^{2}\left(t\right)\right]{\left(\sum_{i=1}^{N}\alpha_{i}\right)}^{2}}{N\sigma^{2}}$$

where E[.] is the expectation operation. After DC bias is removed, the effective SNR per received symbol is [26]

(8) $$\gamma_{\mathrm{EGC}}^{\mathrm{eff}}=\frac{1}{1+g^{2}}\gamma_{EGC}=\frac{\bar{\gamma }{\left(\sum_{i=1}^{N}\alpha_{i}\right)}^{2}}{N}$$

where

(9) $$\bar{\gamma }=\frac{{\left(\eta Ps\right)}^{2}E\left[x^{2}\left(t\right)\right]}{\left(1+g^{2}\right)\sigma^{2}}$$

where $\bar{\gamma }$ denotes the average SNR per symbol. 

At the receiver, using two matched filters that were the time-reversed versions of transmitter filters, y(t) was separated into two orthogonal signals, followed by downsampling, QAM demapping, and LDPC decoding. The transmitted bits were then compared with the decoded bits to estimate the BER.

### 2.2. Underwater-Optical-Turbulence Channel

In this paper, GG statistical distribution was adopted to model the UOT, i.e., the fading coefficient α had a probability density function (PDF) given by [12]

(10) $$f_{\alpha }\left(\alpha \right)=\frac{2\nu }{{\left(\Omega /m\right)}^{m}\Gamma \left(m\right)}\alpha^{2\nu m-1}\exp\left(-\frac{m\alpha^{2\nu }}{\Omega }\right),\;\alpha \geq 0$$

where m, Ω, and ν represent the fading, scaling, and shape parameters, respectively, and Γ(·) is the gamma function defined as Equation (8.310/1) in [27]. 

Scintillation index $\delta_{I}^{2}$ is defined as [8]

(11) $$\delta_{I}^{2}=\frac{E\left[\alpha^{2}\right]-E^{2}\left[\alpha \right]}{E^{2}\left[\alpha \right]}$$

where the first and second moments of α are expressed as [11]:

(12) $$E\left[\alpha \right]=\frac{\Gamma \left(m+\frac{1}{2\nu }\right)}{{\left(\frac{\Omega }{m}\right)}^{2\nu }\Gamma \left(m\right)}$$

(13) $$E\left[\alpha^{2}\right]=\frac{\Gamma \left(m+\frac{1}{\nu }\right)}{{\left(\frac{\Omega }{m}\right)}^{\nu }\Gamma \left(m\right)}$$

Thus, the scintillation index was given by

(14) $$\delta_{I}^{2}=\frac{\Gamma \left(m\right)\Gamma \left(m+\frac{1}{\nu }\right)}{\Gamma^{2}\left(m+\frac{1}{2\nu }\right)}-1$$

To ensure that no energy loss or gain occurs during the turbulence-induced fading process, the fading coefficient was normalised, i.e., E[α]=1. Then, we had

(15) $$\Omega =m{\left(\frac{\Gamma \left(m\right)}{\Gamma \left(m+\frac{1}{2\nu }\right)}\right)}^{2\nu }$$

Optical-intensity fluctuation due to UOT was characterised by multiplicative fading coefficient α [3].

### 2.3. Selection of LDPC Codes 

In this study, we employed a quasicyclic LDPC (QC-LDPC) code based on the IEEE 802.16 standard. Compared to the randomly structured codes, QC-LDPC codes are more advantageous due to lower encoding and decoding complexity, less storage space, more flexibility, and simpler implementation [28]. The output codeword of LDPC code is represented as *c* = (*s*, *p*1, *p*2), where *s* represents systematic bits, and *p*1 and *p*2 are parity-check bits. Detailed techniques for constructing and encoding LDPC can be found in [20,29]; hence, it is not explained in detail here. The output codewords were mapped to *M*-QAM, followed by *M*-CAP modulation and transmission through the UVLC channel as described in the section above. At the receiver end, the electrical signal given in Equation (6) was soft-demapped to provide initial bit likelihoods for belief-propagation (BP) algorithm-based LDPC decoder. When the modulated symbols were equiprobable, the log-likelihood ratio (LLR) for the *i*th bit $b_{i}$ was represented as [30]

(16) $$L\left(b_{i}\right)=\ln\frac{\sum_{b_{i}=0}p\left(y\left(t\right)|x\left(t\right)\right)}{\sum_{b_{i}=1}p\left(y\left(t\right)|x\left(t\right)\right)}$$

where p(y(t)|x(t)) represents the probability of y(t) conditioned on *x*(*t*), which is given by:

(17) $$p\left(y\left(t\right)|x\left(t\right)\right)=\frac{1}{\sigma \sqrt{2\pi N}}\exp\left(\frac{{\left(y\left(t\right)-\eta x\left(t\right)Ps\sum_{i=1}^{N}\alpha_{i}\right)}^{2}}{2N\sigma^{2}}\right)$$

where we used Gaussian approximation in the calculation of symbol reliabilities [13], and assumed that the sum of fading coefficients $\sum_{i=1}^{N}\alpha_{i}$ was known to the receiver by automatic gain controller due to slow fading.

LLRs obtained by soft-demapping *M*-QAM symbols were fed into the LDPC decoder as a priori inputs. An iterative decoding min–sum algorithm (MSA) [31] based on a soft decision was utilised for LDPC decoding. The MSA is a lower-complexity approximation to the sum-product algorithm (SPA), and can provide better BER performance than the bit-flipping (BF) algorithm based on the hard decision. 

## 3. Approximated BER without FEC

CAP and QAM have the same BER in theory. An approximated conditional bit-error probability (BEP) of *M*-QAM is given by [32]

(18) $$p\left(\varepsilon |\gamma \right)=\frac{4\left(\sqrt{M}-1\right)}{\sqrt{M}\log_{2}M}Q\left(\sqrt{\frac{3}{M-1}\gamma }\right)+\frac{4\left(\sqrt{M}-2\right)}{\sqrt{M}\log_{2}M}\;Q\left(3\sqrt{\frac{3}{M-1}\gamma }\right)$$

where Q(·) is the Gaussian Q-function, and γ is the electrical SNR. The average BER can be obtained by integrating BEP over $p_{\gamma }\left(\gamma \right)$, namely,

(19) $$P_{b}=\int_{0}^{\infty }p\left(\varepsilon |\gamma \right)p_{\gamma }\left(\gamma \right)d\gamma$$

However, the integration in Equation (19) was complicated, as it was very difficult to replace the multidimensional integral resulting from $p_{\gamma }\left(\gamma \right)$ with an equivalent single-fold integral. So, we adopted the CHF-based approach proposed in [33] to derive analytical expressions for the BER of CAP with the EGC diversity scheme over the GG fading channels. The expressions depend on the single-fold integral, and can be presented in the form of Fox’s H-function or Meijer’s G-function, which greatly simplify the calculations.

Using the alternative form of Q(·) as shown as Equation (4.2) in [34], and according to Equations (2), (7) and (9) in [33], the approximated BER can be expressed as follows:

(20) Pb=1π∫0∞Re{φb(w)ϕγEGCeff*(w)}dw

where w is the angular frequency, $\varphi_{b}\left(w\right)$ is the CHF of the conditional BEP $p\left(\varepsilon |\sqrt{\gamma_{\mathrm{EGC}}^{\mathrm{eff}}}\right)$, and $\phi_{\sqrt{\gamma_{\mathrm{EGC}}^{\mathrm{eff}}}}^{*}\left(w\right)$ is the complex conjugate of the CHF of $\sqrt{\gamma_{\mathrm{EGC}}^{\mathrm{eff}}}$. The real and imaginary parts of $\varphi_{b}\left(w\right)$ are, respectively, given by [35]

(21) Re{φb(w)}=12∑z=1Z∫0θzaz(θ)πsin2θϑzexp(−w2sin2θ4ϑz)dθIm{φb(w)}=12∑z=1Z∫0θzaz(θ)wsin2θϑz 1F1(1;32;−w2sin2θ4ϑz)dθ

where *_1_F_1_* (·) is the confluent hypergeometric function of the first kind shown as Equation (9.210/1) in [27], and parameters Z, $a_{z}$ $\theta_{z}$, and $\vartheta_{z}$ for *M*-QAM are given in Table 1. 

Then, when we derive $\phi_{\sqrt{\gamma_{\mathrm{EGC}}^{\mathrm{eff}}}}\left(w\right)$. From Equation (8), we have

(22) $$\sqrt{\gamma_{\mathrm{EGC}}^{\mathrm{eff}}}=\sqrt{\frac{\bar{\gamma }}{N}}\overset{N}{\underset{i=1}{\sum }}\alpha_{i}$$

Assuming the receiver separations are higher than underwater coherence distance, the fading coefficients are independent; hence, the CHF of Equation (22) is given as

(23) $$\phi_{\sqrt{\gamma_{\mathrm{EGC}}^{\mathrm{eff}}}}\left(w\right)=\overset{N}{\underset{i=1}{\prod }}\phi_{\alpha_{i}}\left(\sqrt{\frac{\bar{\gamma }}{N}}w\right)$$

where $\phi_{\alpha_{i}}\left(w\right)$ is the CHF of $\alpha_{i}$ that can be obtained using the approach detailed in [35]. Using Equation (10), CHF definition, Euler’s formula, the contour integrals of sine and cosine function [36], the definition of the gamma function, and Fox’s H function given by Equation (1.2) in [37], we obtained the real part of $\phi_{\alpha_{i}}\left(w\right)$ as

(24) Re{ϕαi(w)}=πΓ(mi)12πj∫ϱ−j∞ϱ+j∞((w2)2(Ωimi)1νi)sΓ(−s)Γ(mi+sνi)Γ(12+s)ds=πΓ(mi)H1,21,1[((w2)2(Ωimi)1νi)|(1−mi,1/vi)(0,1),(1/2,1)]

where j is the imaginary unit, ϱ is a constant chosen in the region of convergence on the complex plane, and $H_{p,q}^{e,f}$*[*·*]* is Fox’s H function. Similarly, the imaginary part of $\phi_{\alpha_{i}}\left(w\right)$ is obtained as

(25) Im{ϕαi(w)}=πΓ(mi)H1,21,1[((w2)2(Ωimi)1νi)|(1−mi,1/vi)(1/2,1),(0,1)]

Substituting Equations (21), (24) and (25) in Equation (20), we obtained a closed-form expression in the form of Fox’s H function for the approximated BER of *M*-QAM with EGC diversity over GG fading channels. The BER of CAP was efficiently calculated on the basis of MATHEMATICA implementation for Fox’s H function presented in [38].

## 4. Results and Discussion

In this section, the BER performance of the proposed LDPC-coded CAP with spatial-diversity UVLC system was evaluated using a Monte Carlo simulation under different turbulence conditions and diversity orders. Meanwhile, the analytical BER of uncoded CAP with spatial-diversity UVLC system (given by Equation (20)) was verified by comparing analytical and simulation results. The simulation parameters for this study are given in Table 2. 

**Figure 2:**
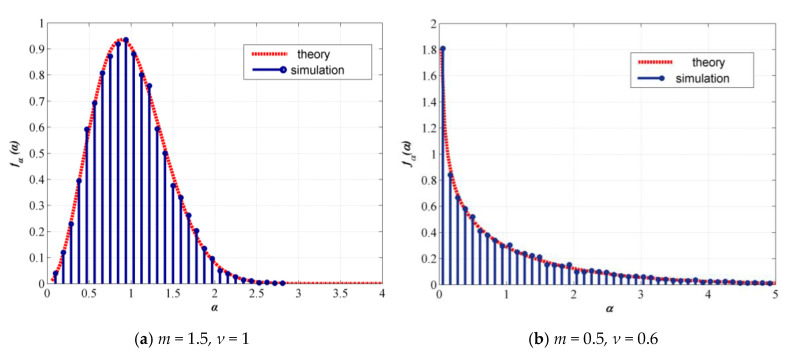
Probability-density function (PDF) of GG distribution.

First, the theoretical and simulated PDFs of the GG distribution are presented in Figure 2, showing good agreement, hence ensuring accurate GG turbulence channels for BER estimation.

**Figure 3:**
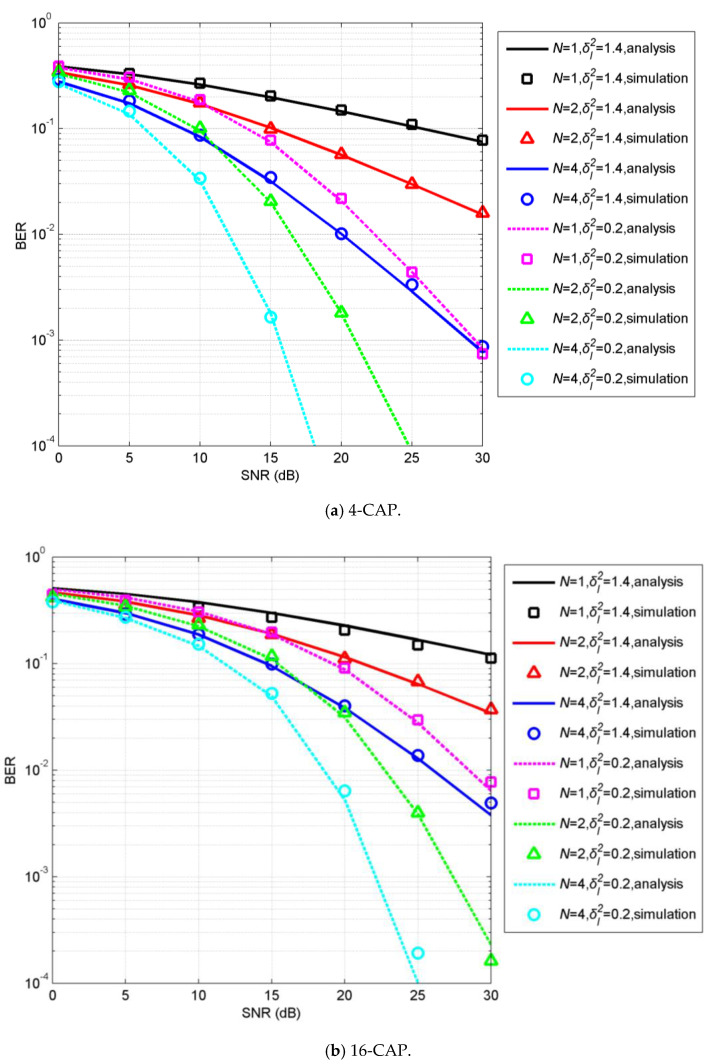
Bit-error rates (BERs) of uncoded 4- and 16-carrierless amplitude and phase (CAP) with spatial diversity underwater-visible-light-communication (UVLC) systems over turbulent channels.

Figure 3 shows the BER performance of uncoded CAP with spatial diversity over GG turbulence channels, where we assumed subchannels were independent of each other. For comparison, results for the system without diversity (i.e., *N* = 1) are also presented. Figure 3 shows that spatial diversity was effective in mitigating the adverse turbulence effect. Furthermore, analytical BERs approximately matched simulated BERs, validating the analysis. Obviously, BERs increase with increasing constellation size and turbulence strength. A larger diversity order or power penalty is required for 16-CAP to obtain the same BER performance as that of 4-CAP. For example, in weak turbulence channel with $\delta_{I}^{2}=0.2$, 16-CAP with *N* = 2 needed an additional SNR of 6.5 dB to obtain a BER of 10^−3^ compared to 4-CAP. Additionally, to achieve a BER of 10^−4^ at the SNR of ~25 dB, diversity orders were 2 and 4 for 4-CAP and 16-CAP, respectively. Thus, a trade-off between spectral efficiency and reliability should be carefully considered in practical implementation. 

**Figure 4:**
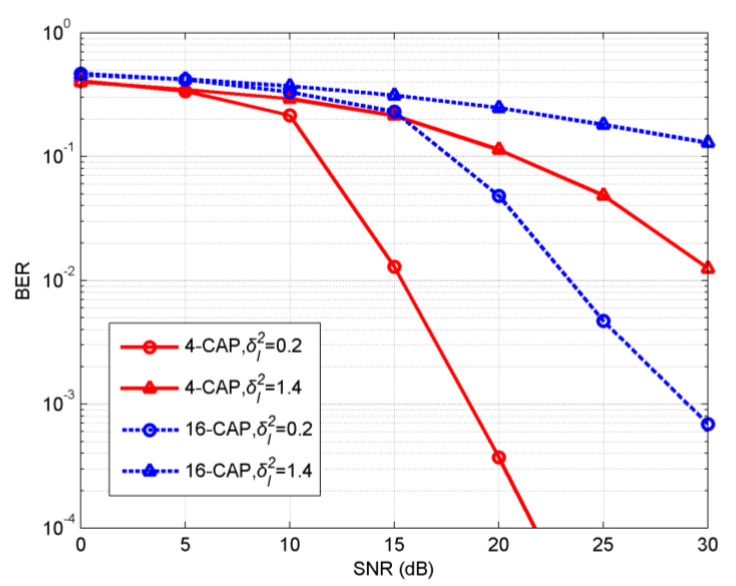
BERs of LDPC-coded 4- and 16-CAP single-branch UVLC over turbulent channels.

To obtain further performance improvement, we studied the effect of LDPC to mitigate fading on the basis of MC simulations. The BER performance of LDPC-coded 4- and 16-CAP without diversity in a GG turbulence UVLC channel is shown in Figure 4. LDPC codes were effective in mitigating the adverse effect of turbulence for the 4- and 16-CAP systems over a weak turbulence channel with $\delta_{I}^{2}=0.2$. For example, using LDPC codes, a waterfall of BER of 4-CAP was achieved at the SNR of ~24 dB and the BER of 16-CAP fall by an order of magnitude at the SNR of 30 dB. However, LDPC codes did not provide significant improvement over a strong turbulence channel with $\sigma_{I}^{2}=1.4$. That is because the performance of LDPC-coded schemes depends on the input of the LDPC decoder, i.e., soft-demapping information. Hence, the performance of a coded CAP scheme with spatial diversity was studied to mitigate the turbulence effect. 

**Figure 5:**
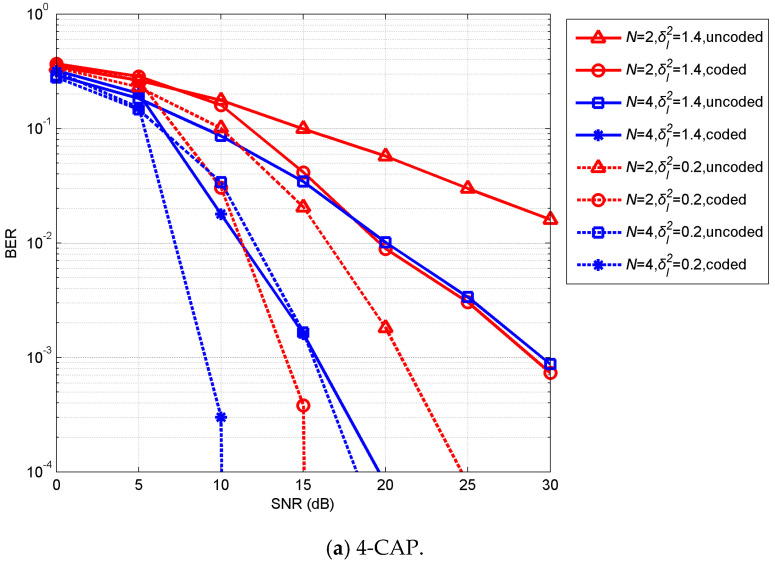

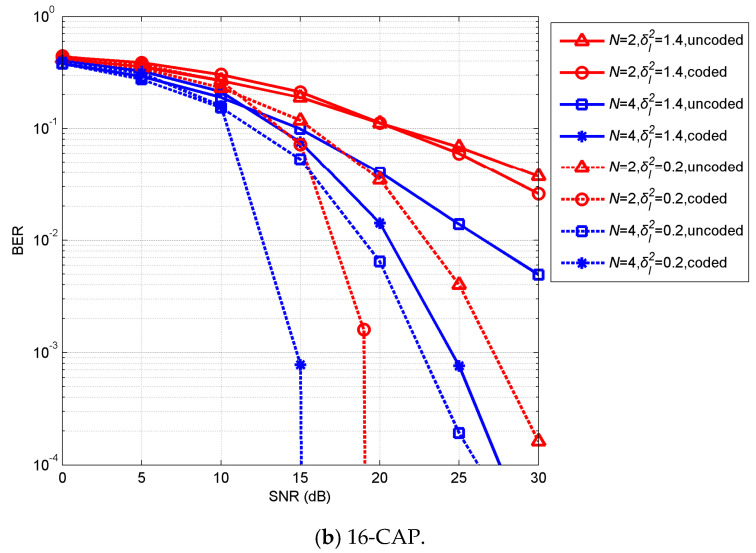
BERs of LDPC-coded CAP with spatial diversity UVLC systems over turbulent channels.

Figure 5 shows the BER performance of the coded CAP with spatial diversity under turbulent UVLC channels. In weak turbulence with $\delta_{I}^{2}=0.2$, the coded spatial-diversity scheme with *N* = 2 had better performance than that of the spatial-diversity-only scheme with *N* = 4, resulting in BER waterfalls at SNRs of ~15 and ~19 dB for 4- and 16-CAP, respectively. In strong turbulence with $\sigma_{I}^{2}=1.4$, the coded spatial-diversity scheme with *N* = 4 could obtain high coding gain and achieve a BER of 10^−4^ at SNRs of ~19.5 and ~27.5 dB for 4- and 16-CAP, respectively, while even at the SNR of 30 dB, the BERs of the coded-only systems were larger than 10^−2^ (shown in Figure 4), and the BERs of the diversity-only systems with *N* = 4 were larger than 9 × 10^−4^. So, by comparing Figure 4 and Figure 5, LDPC codes needed to combine with spatial diversity to combat the strong turbulence, and were helpful in achieving a desired BER with lower received optical power compared to uncoded systems. Received optical power is limited by transmitter and attenuation, and fluctuated by turbulence, so the combination of LDPC and spatial diversity is very useful for UVLC. 

## 5. Conclusions

In this paper, we studied the performance of LDPC-coded CAP with spatial diversity for UVLC. Expressions for approximated BERs were derived for systems without FEC code. Furthermore, Monte Carlo simulation was used to verify the analytical study of the uncoded system. We showed that the diversity scheme was effective in mitigating the effect of turbulence, and further studied the performance of the LDPC-coded scheme with and without diversity over turbulent channels. Obtained results showed that the LDPC-coded scheme without diversity was not enough to combat strong turbulence, while spatial diversity with LDPC codes offered the best performance. In turbulence with $\sigma_{I}^{2}=1.4$, the BER of the CAP UVLC system without coding and diversity was close to 10^−1^ at the SNR of 30 dB, while the LDPC-coded CAP with a spatial-diversity system with *N* = 4 achieved a BER of 10^−4^ around SNRs of 19.5 and 27.5 dB for 4- and 16-CAP, respectively. Results demonstrated that the effect of turbulence on BER performance could be effectively mitigated by spatial diversity and LDPC codes.

## Figures and Tables

**Table 1:** *M*-quadrature-amplitude-modulation (*M*-QAM) parameters.

Parameters	Z	$a_{z}$	$\vartheta_{z}$	$\theta_{z}$
Value	2	$a_{1}=\frac{4}{\pi }\left(\frac{\sqrt{M}-1}{\sqrt{M}\log_{2}M}\right)$ $a_{2}=\frac{4}{\pi }\left(\frac{\sqrt{M}-2}{\sqrt{M}\log_{2}M}\right)$	$\vartheta_{1}=\frac{3}{2\left(M-1\right)}$ $\vartheta_{2}=\frac{27}{2\left(M-1\right)}$	$\theta_{1}=\theta_{2}=\frac{\pi }{2}$

**Table 2:** Simulation parameters. Note: GG, generalized-gamma; LDPC, low-density parity check.

Parameter	Value
*M*	4, 16
Pseudorandom bits length (PRBS)	>2^17^
Symbol duration	4 × 10^−8^ s
Roll factor, β	0.15
Upsampling factor	20
Carrier frequency: symbol rate × (1 + β)/2	14.375 MHz
Normalised DC bias	3
GG-distributed parameters (*m, ν*)	(1.5,1), (0.5,0.6)
Scintillation index	0.2,1.4
LDPC code length and rate	2304, 1/2
Diversity order	1, 2, 4
